# GSK-3Beta-Dependent Activation of GEF-H1/ROCK Signaling Promotes LPS-Induced Lung Vascular Endothelial Barrier Dysfunction and Acute Lung Injury

**DOI:** 10.3389/fcimb.2017.00357

**Published:** 2017-08-04

**Authors:** Lei Yi, Xiaoqin Huang, Feng Guo, Zengding Zhou, Mengling Chang, Jingning Huan

**Affiliations:** ^1^Department of Orthopedics, Shanghai Fengxian Central Hospital, Shanghai University of Medicine and Health Sciences Affiliated Sixth People's Hospital South Campus Shanghai, China; ^2^Department of Burn and Plastic Surgery, Ruijin Hospital, School of Medicine, Shanghai Jiao Tong University Shanghai, China

**Keywords:** lipopolysaccharide, endothelial cell, ALI, GSK-3beta, GEF-H1, ROCK

## Abstract

The bacterial endotoxin or lipopolysaccharide (LPS) leads to the extensive vascular endothelial cells (EC) injury under septic conditions. Guanine nucleotide exchange factor-H1 (GEF-H1)/ROCK signaling not only involved in LPS-induced overexpression of pro-inflammatory mediator in ECs but also implicated in LPS-induced endothelial hyper-permeability. However, the mechanisms behind LPS-induced GEF-H1/ROCK signaling activation in the progress of EC injury remain incompletely understood. GEF-H1 localized on microtubules (MT) and is suppressed in its MT-bound state. MT disassembly promotes GEF-H1 release from MT and stimulates downstream ROCK-specific GEF activity. Since glycogen synthase kinase (GSK-3beta) participates in regulating MT dynamics under pathologic conditions, we examined the pivotal roles for GSK-3beta in modulating LPS-induced activation of GEF-H1/ROCK, increase of vascular endothelial permeability and severity of acute lung injury (ALI). In this study, we found that LPS induced human pulmonary endothelial cell (HPMEC) monolayers disruption accompanied by increase in GSK-3beta activity, activation of GEF-H1/ROCK signaling and decrease in beta-catenin and ZO-1 expression. Inhibition of GSK-3beta reduced HPMEC monolayers hyper-permeability and GEF-H1/ROCK activity in response to LPS. GSK-3beta/GEF-H1/ROCK signaling is implicated in regulating the expression of beta-catenin and ZO-1. *In vivo*, GSK-3beta inhibition attenuated LPS-induced activation of GEF-H1/ROCK pathway, lung edema and subsequent ALI. These findings present a new mechanism of GSK-3beta-dependent exacerbation of lung micro-vascular hyper-permeability and escalation of ALI via activation of GEF-H1/ROCK signaling and disruption of intracellular junctional proteins under septic condition.

## Introduction

Sepsis is a serious consequence of Gram-negative infection, which accounts for high mortality in critically ill patients (Wendel et al., 2007). Exacerbated inflammation and derangement of coagulation responses are hallmarks of acute lung injury (ALI) and acute respiratory distress syndrome (ARDS) (Yi et al., 2016). Along with acute alterations in blood hyper-coagulation and inflammatory activation of lung vascular cells, lung vascular endothelial barrier dysfunction played a more important role in LPS-induced lung edema and ALI. The permeability of endothelial cells (EC) monolayer is mainly controlled by cell-cell contact protein complexes which contain adherens junctions (AJs) and tight junctions (TJs) (Dejana, 2004). The TJ component zona occludens-1 (ZO-1) and AJ component beta-catenin are special intracellular junctional proteins, which associate with many transmembrane proteins and cytoskeletal binding proteins (Stevens et al., 2000). Besides acting as adaptors in mediating the binding of adhesion proteins to actin, ZO-1 and beta-catenin function as scaffolds not only for stabilization of the junctions, but also for the maintenance of ECs shape and polarity (Dudek and Garcia, 2001; Lampugnani et al., 2002). Therefore, lung micro-vascular barrier compromise is associated with disruption of ZO-1 and beta-catenin.

Guanine nucleotide exchange factor H1 (GEF-H1) is a Rho-specific GEF, which localizes on microtubules (MT). The guanine exchange activity of GEF-H1 is suppressed in its MT-bound state. After MTs disassembly, GEF-H1 release from MT and stimulates RhoA-specific GEF activity (Krendel et al., 2002). We have recently reported that LPS rapidly induced MT disassembly in ECs (Zhou et al., 2015). Moreover, we found that GEF-H1 plays an important role in Rho activation in ECs exposed to LPS and vascular barrier dysfunction (Zhou et al., 2013). Previous studies also shown that GEF-H1-mediated activation of ROCK positively regulates LPS-induced lung vascular leak *in vivo*, which is caused by LPS-induced MT disassembly (Kratzer et al., 2012). In addition, MT stabilization could protect against thrombin-induced GEF-H1/ROCK signaling activation and vascular barrier compromise (Birukova et al., 2006). However, the mechanisms responsible for the MT-mediated GEF-H1/ROCK pathway activation during LPS-induced lung vascular hyper-permeability and ALI have not been well characterized.

Glycogen synthase kinase (GSK-3beta) is a serine threonine protein kinase implicated in regulating intracellular signaling pathway associated with cell metabolism, proliferation and apoptosis (Cohen and Frame, 2001; Kaidanovich-Beilin and Woodgett, 2011). Although originally described as a regulator of glycogen synthase involved in the development of diabetes and cancer (Ali et al., 2001; Luo, 2009), GSK-3beta plays a significant role in modulating the development of sepsis and shock (Dugo et al., 2007). For instance, GSK-3beta inhibitors restrict the severity of ALI by preventing the alveolar epithelial cells death (Guo et al., 2014). Inhibiting GSK-3beta functional activity attenuates endotoxin-induced liver injury (Gong et al., 2013). Reducing the activity of GSK-3beta inhibits LPS-induced tissue factor induction and deep vein thrombosis (Dong et al., 2015). Blockade of GSK-3beta activation significantly reduces LPS-induced mortality in mice and diminishes pro-inflammatory cytokine production in LPS-treated monocytes (Martin et al., 2005). In addition to modulating the magnitude of inflammatory conditions, GSK-3beta has also been involved in suppressing the production of anti-inflammatory cytokines (Chan et al., 2009). However, the effects of GSK-3beta on lung micro-vascular barrier disruption under sepsis remain to be defined.

Interestingly, previous studies suggested that GSK-3beta could orchestrate MT remodeling (Xu et al., 2015). Cigarette smoke disrupted lung vascular endothelial barrier and increased susceptibility to ALI via GSK-3beta activation and subsequent MT disassembly (Borgas et al., 2016). Moreover, it has been reported that inactivation of GSK-3beta remarkably inhibited RhoA activation, stress fiber formation and cancer cell migration (Liu et al., 2013). As we all know, the canonical Wnt signaling pathway is the classical mechanism of GSK-3beta-induced degradation of beta-catenin. Without Wnt stimulation, beta-catenin in the cytoplasm is constitutively targeted for degradation by the destruction complex consisting of adenoma polyposis coli (APC), axin, glycogen synthase kinase 3 (GSK-3), and casein kinase 1 (CK1) (Liu et al., 2002). Interestingly, beta-catenin not only acts as a transcription factor of Wnt signaling pathway but also serves as a significant endothelial cell-cell junctional protein which maintains the integrity of endothelial cell barrier. When endothelial cell contacted each other and formed monolayer, the beta-catenin detached from APC-mediated destruction complex in cytoplasm and was bound to endothelial AJs and further stabilized and retained at the endothelial cell membrane (Hulsken et al., 1994). Amy Barton-Pai et al found that Tumor Necrosis Factor-a induces lung vascular hyper-permeability via the activation of GSK-3beta and subsequent decrease of un-phospho-beta-catenin (Ser33/37) (Barton-Pai et al., 2011). In addition, GSK-3beta inhibitor increases the stabilization of TJ complex at the Blood-Brain Barrier (BBB) by regulating ZO-1 under physiologic conditions (Ramirez et al., 2013). Given the role of GSK-3beta in regulating dynamics of MT and in regulating junctional proteins, we examined whether GSK-3beta-dependent activation of GEF-H1/ROCK signaling promotes the disruption of beta-catenin and ZO-1 in lung vascular endothelial cell monolayers *in vitro* as well as increases lung vascular leak and ALI *in vivo* under septic conditions.

In the present study, we showed that LPS-induced activation of GSK-3beta/GEF-H1/ROCK signaling contributed to disruption of ZO-1 and beta-catenin in HPMECs. Moreover, inhibition of GSK-3beta not only decreased the expression of GEF-H1 and ROCK in lung lysate but also alleviated LPS-induced lung edema and ALI. Our findings give us a novel insight into the pathogenic mechanisms of LPS-induced lung micro-vascular hyper-permeability, which may be a new therapeutic direction for ALI under septic conditions.

## Materials and methods

### Cell culture and reagents

Primary human pulmonary micro-vascular endothelial cells (HPMECs) were obtained ScienCell Research Laboratories and maintained in ScienCell Endothelial Cell Medium in a humidified 37°C, 5% CO_2_ incubator. The primary cells were used at passages 6–8. The medium was changed at 48-h intervals. The LPS (from Escherichia coli 055:B5), Y-27632 and SB-216763 were purchased from Sigma-Aldrich (St. Louis, MO). The anti-GEF-H1 rabbit monoclonal antibody (mAb), anti-phospho-myosin-associated phosphatase type 1 (MYPT1) (Thr696) rabbit mAb, anti-beta-catenin rabbit mAb, and anti-rabbit immunoglobulin-G-HRP-linked antibody were obtained from Cell Signaling Technologies (Danvers, Mass). The anti-ZO-1 goat polyclonal Ab, anti-phospho-beta-catenin (Ser33/37) mouse mAb, anti-phospho-GSK-3β (Tyr 216) rabbit polyclone Ab, anti-GSK-3β mouse mAb, anti-mouse immunoglobulin-G-HRP-linked antibody and anti-goat immunoglobulin-G-HRP-linked antibody were purchased from Santa Cruz Biotechnology (Santa Cruz, CA). The Alexa Fluor 488-conjugated donkey anti-Goat secondary antibody and Alexa Fluor 488-conjugated goat anti-rabbit secondary antibody and the ProLong Gold Anti-fade Mountant with DAPI were obtained from Invitrogen Life Science.

### Measurement of barrier permeability by trans-endothelial electrical resistance (TER)

The measurement of HPMEC monolayers TER was performed by the electric cell-substrate impedance system (ECIS) as previously described (Fu et al., 2015). The HPMECs were plated on gelatin-coated 8W10E electrode arrays at 1 × 10^5^/well, and the ECs were allowed to form tight monolayers until stable TER values were reached. The monolayers were exposed to the indicated GSK-3β inhibitor before the stimulation with LPS and the changes in TER was monitored continuously by the ECIS system. The results were acquired at 4,000 Hz.

### Western blot assay

The total protein was extracted from HPMEC monolayers or lung tissue extracts, and then the target proteins expression were probed with specific antibodies. The equal amounts of cells or lung tissue extracts were separated by 6–12% sodium dodecyl sulfate polyacrylamide gel electrophoresis (SDS-PAGE) according to the molecular weight of the target proteins and electro-transferred to PVDF membrane. The membranes were blocked in Tris-buffered saline (TBS) and Tween-20 containing 5% nonfat milk at room temperature for 1 h at room temperature and then incubated with antibodies to the target proteins (GSK-3β, P-GSK-3β (Tyr216), P-beta-catenin (Ser33/37) and ZO-1 dilutions were 1:500; GEF-H1, P-MYPT1 (Thr696) and beta-catenin dilutions were 1:1,000) overnight at 4°C. The membranes were incubated with the appropriate HRP-linked secondary antibodies (1:2,000) at room temperature for 1 h. The signal intensities were compensated by glyceraldehyde 3- phosphate dehydrogenase (GAPDH) as internal controls. Finally, the bands were developed with western blot luminal reagent (Millipore, Billerica, MA). Protein bands were quantified using ImageQuantR software (Molecular Dynamics, Sunnyvale, CA).

### Transfection with GEF-H1 siRNA *in vitro*

Stealth RNA interference duplexes against human GEF-H1 were designed and synthesized by Gene pharma Technologies (Shanghai, China). GEF-H1 siRNA molecules were transfected individually into HPMECs using Lipofectamine RNAiMAX (Carlsbad, CA) according to the manufacturer's instructions. Duplex siRNA was constructed against sequences coding for GEF-H1 positions 998–1,022 (5′-AGA ACU GGC UGA UGA GCA GAU CAC C-3′). A scrambled, negative control siRNA (5′-UUC UCC GAA CGU GUC ACG UTT-3′) was also included. The ability of RNA interference molecules to knockdown target protein expression was analyzed by western blot analysis. Transfections of FITC-labeled nonspecific siRNA revealed that transfection efficiency was up to 85% in HPMECs.

### Confocal immunofluorescent analysis

For beta-catenin and ZO-1 staining, confluent HPMECs monolayers were washed twice with pre-warmed PBS (pH 7.4), fixed in 4% paraformaldehyde for 15 min and then permeabilized with PBS containing 0.1% Triton X-100 and 1% bovine serum albumin for 10 min. After washing in PBS, the monolayers were incubated with the anti-beta-catenin rabbit mAb (1:100) and anti-ZO-1 goat polyclonal Ab (1:200) overnight at 4°C. The HPMECs stained with ZO-1 or beta-catenin were further incubated with the Alexa Fluor 488-conjugated donkey anti-Goat secondary antibody (1:500) or Alexa Fluor 488-conjugated goat anti-rabbit secondary antibody (1:500) respectively for 1 h. Finally, HPMECs were sealed by ProLong Gold Anti-fade Mountant with DAPI, and then, the monolayers were imaged with confocal microscopy.

### Animal studies (ali model, lung histopathology, ratios of wet/dry, and evans blue extravasation)

Male C57BL/6 mice (6–8 weeks old, 18–20 g) were obtained from the Experimental Animal Center of Ruijin Hospital, Shanghai, China. All procedures and animal care were performed in accordance with the National Institutes of Health *Guide for the Care and Use of Laboratory Animals* with the approval (SYXK-2011-0113) of the Scientific Investigation Board of Shanghai Jiao Tong University School of Medicine, Shanghai, China. The animals were acclimatized to the laboratory conditions (25°C, 12 h/12 h light/dark, 50% humidity, and *ad libitum* access to food and water) for 1 week prior to experimentation. The mice were firstly pretreated with or without SB-216763 (20 mg/kg) by intraperitoneal injection for 2 h, and then the ALI was induced by an intra-tracheal instillation of LPS at 2 mg/kg for 24 h as previously described (Park et al., 2014), the mice were anesthetized with 1% sodium pentobarbital (40 mg/kg). For histological assessment of lung injury, the lungs were quickly removed and fixed in 10% paraformaldehyde. The paraformaldehyde-fixed lobe of the lungs were embedded in paraffin and cut into 5-μm sections. H&E staining was performed using standard protocols. The slides of each group were assessed under high-power fields (Sections were evaluated at × 100 magnificence). For assessment of LPS-induced lung edema, the lungs were immediately weighed to obtain the wet weight, and then placed in an oven at 80°C for 48 h to obtain the dry weight. The ratio of the wet lung to the dry lung was calculated to assess lung edema. For analysis of LPS-induced lung vascular leak, Evans blue dye (30 ml/kg) was injected into the caudal vein 2 h before termination of the experiment. Measurement of Evans blue accumulation in the lung tissue was performed by spectrofluorimetric analysis of lung tissue lysates as before (Moitra et al., 2007).

### Statistical analysis

The results are expressed as the means ± SEM of at least 3 independent experiments. Data are expressed as mean and standard error. Student's *t*-tests and ANOVAs were used as appropriate. The significance was accepted at *P* < 0.05.

## Results

### LPS induces activation of GSK-3beta in dose- and time-dependent manners in HPMECs

**Figure 1:**
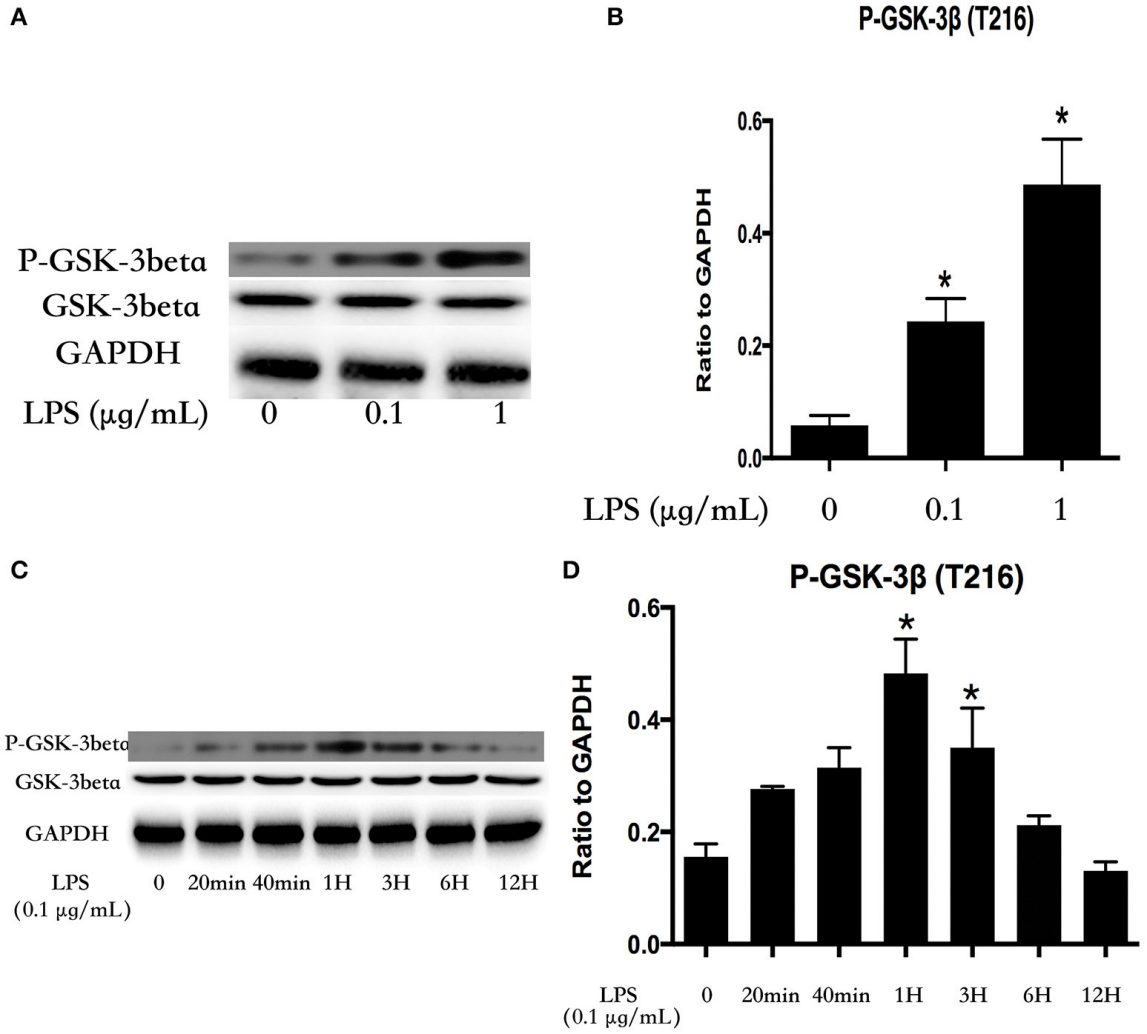
LPS induces GSK-3beta activation in dose- and time-dependent manners in HPMECs. Expression of P-GSK-3beta and GSK-3beta was detected after incubation with different concentrations of LPS for 1 h **(A)**. The expression of P-GSK-3beta was represented as a histogram according to band intensities **(B)**. Expression of P-GSK-3beta and GSK-3beta was examined at indicated time points after stimulation with LPS (0.1 μg/ml) in HPMECs **(C)**. The Western blotting results are presented as a histogram showing the band intensity values **(D)**. ^*^P < 0.05 vs. LPS un-treatment group.

HPMECs were stimulated with various concentrations of LPS (0, 0.1, and 1 μg/ml) for 1 h. GSK-3beta is activated via phosphorylation of GSK-3beta on Tyr216 (Jope and Johnson, 2004), and the expression of P-GSK-3beta (Tyr216) exhibited an increasing trend with alteration of the concentration of LPS (Figures 1A,B). Subsequently, HPMECs were incubated with LPS (0.1 μg/ml) at different indicated times. Immunoblot analysis revealed that LPS increased P-GSK-3beta (Tyr216) expression in a time-dependent manner, and the peak of P-GSK-3beta (Tyr216) expression appeared at 1 h after LPS stimulation. However, there was no significance in P-GSK-3beta (Tyr216) expression between 6 and 12 h after LPS stimulation (Figures 1C,D).

### GSK-3beta regulates LPS-induced GEF-H1/ROCK signaling activation

**Figure 2:**
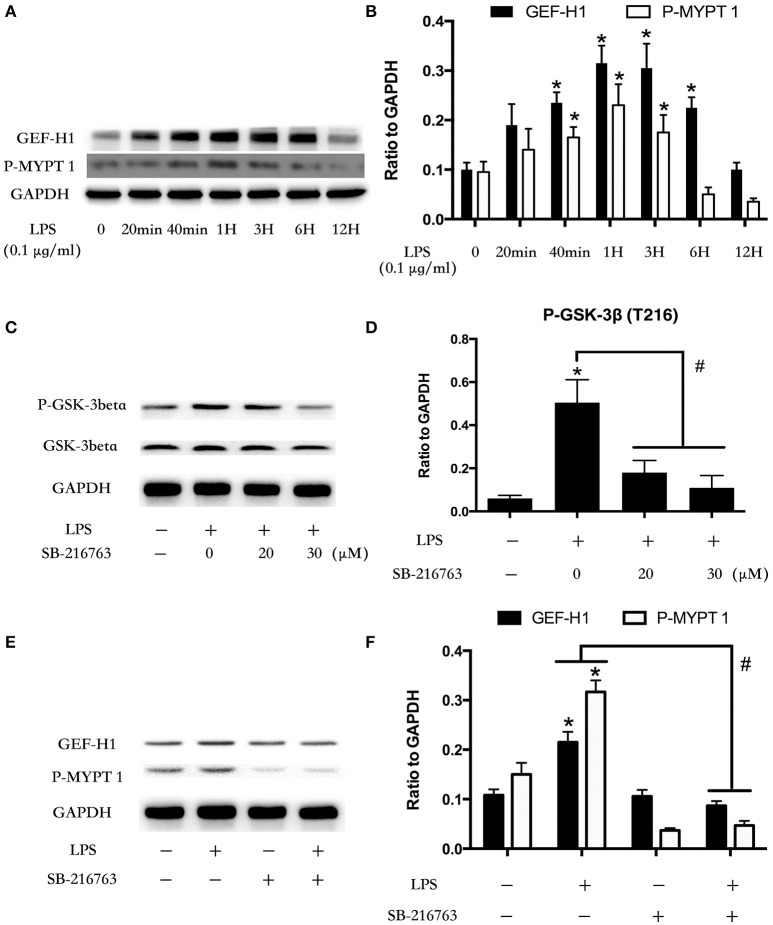
Involvement of GSK-3beta in LPS-induced GEF-H1/ROCK signaling activation. HPMECs were incubated with LPS (0.1 μg/ml) at different indicated times, and the GEF-H1 and myosin-associated phosphatase type 1 (P-MYPT 1: the substrate of ROCK) were detected by Western blot assay **(A)**. The expression of GEF-H1 and P-MYPT 1 were represented as a histogram according to band intensities **(B)**. ^*^ < 0.05 vs. LPS un-treatment group. Inhibition effect of GSK-3beta activity in HPMECs was analyzed by Western blot **(C,D)**. ^*^P < 0.05 vs. the negative control group, ^#^*P* < 0.05 vs. the corresponding LPS treatment group. HPMECs were pretreated with SB-216763 (20 μM) for 1 h and then were exposed to LPS (0.1 μg/ml) for 1 h. The expression of GEF-H1 and P-MYPT 1 were determined by Western blot **(E)**. The Western blotting results are presented as a histogram showing the band intensity values **(F)**. ^*^P < 0.05 vs. the negative control group, ^#^*P* < 0.05 vs. the corresponding LPS treatment group.

Microtubule was the downstream of GSK-3beta (Xu et al., 2015). LPS stimulation rapidly mediated microtubule de-polymerization and subsequent activation of GEF-H1/ROCK pathway in HUVECs (Zhou et al., 2013, 2015). So we further investigated the relationship between GSK-3beta and GEF-H1/ROCK in LPS-stimulated HPMECs. We firstly explored the expression of GEF-H1 and ROCK activity after treating with LPS at indicated time points in HPMECs. The activity of ROCK was analyzed by assessing the phosphorylation of its substrate MYPT 1 (Mali et al., 2011). Interestingly, the LPS-induced activation of GEF-H1/ROCK exhibited a similar trend with the activation of GSK-3beta in HPMECs (Figures 2A,B). Some previous studies have reported the activity of GSK-3beta could be inhibited by SB216763 (Bellei et al., 2008; Mozaffari and Schaffer, 2008; Liu et al., 2015; Chen et al., 2016). Subsequently, we used SB216763 to analyze the effect of GSK-3beta on LPS-induced GEF-H1/ROCK activation. We pretreated the HPMECs with different concentrations of SB-216763 (the specific inhibitor of GSK-3beta) (0, 20, and 30 μM) for 1 h before LPS stimulation. We observed that 20 μM SB-216763 could effectively block the activation of GSK-3β in LPS-stimulated HPMECs (Figures 2C,D). Subsequently, the HPMECs were pretreated with SB-216763 (20 μM) for 1 h and then incubated with LPS (0.1 μg/ml) for another 1 h. We found that SB-216763 effectively inhibited the LPS-induced GEF-H1/ROCK pathway activation (Figures 2E,F).

### Role of GSK-3beta in LPS-induced HPMEC monolayers barrier disruption

**Figure 3:**
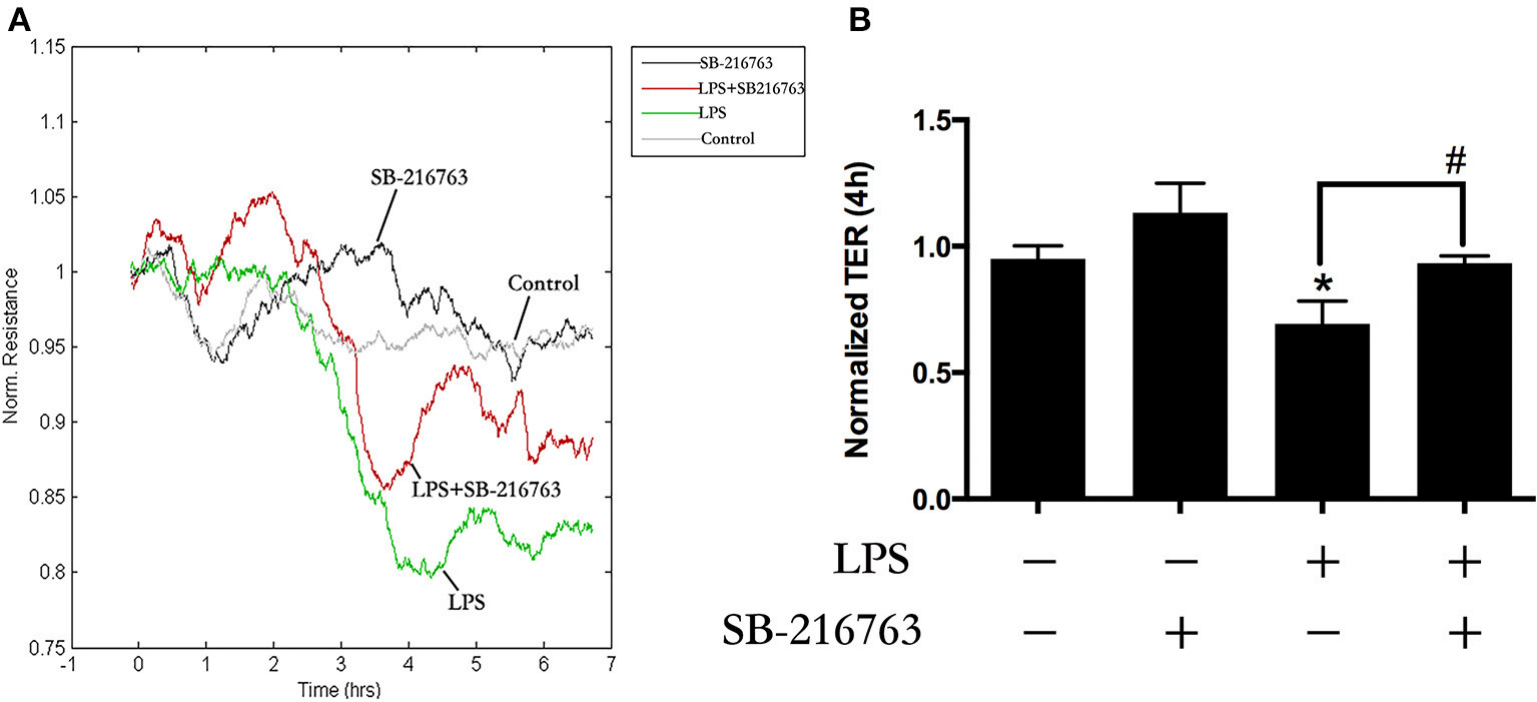
GSK-3beta signaling is involved in LPS-induced HPMECs barrier disruption. The HPMECs were plated on the gold microelectrodes. When HPMECs formed monolayers and reached stable TER values, the SB-216763 (20 μM) was added. After 1 h, the medium or LPS (0.1 μg/ml) was added for another 6 h. The HPMEC monolayers permeability was determined by real-time TER measurement **(A)**. The results of the 3 h LPS stimulation were represented as a histogram in **(B)** according to the TER curves. ^*^P < 0.05 vs. negative control. ^#^*P* < 0.05 vs. corresponding LPS-stimulated group.

Our previous study found that LPS induced EC monolayers Evans blue-labeled albumin (EB-albumin) leak via the activation of GEF-H1/ROCK signaling (Zhou et al., 2013). To further investigate the underlying roles of GSK-3beta on integrity of HPMECs barrier after stimulation of LPS, we incubated ECs with SB-216763 (20 μM) for 1 h and then added LPS (0.1 μg/ml). Consistent with the previous studies (Birukova et al., 2015), LPS rapidly induced ECs barrier disruption and decrease of trans-endothelial electrical resistance (TER). However, pretreatment with SB-216763 effectively revised the LPS-induced TER decrease in HPMEC monolayers (Figures 3A,B). These results suggested that the GSK-3beta/GEF-H1/ROCK signaling is involved in LPS-induced HPMECs barrier dysfunction.

### GSK-3beta/GEF-H1/ROCK pathway is involved in LPS-induced TJs and AJs disruption

**Figure 4:**
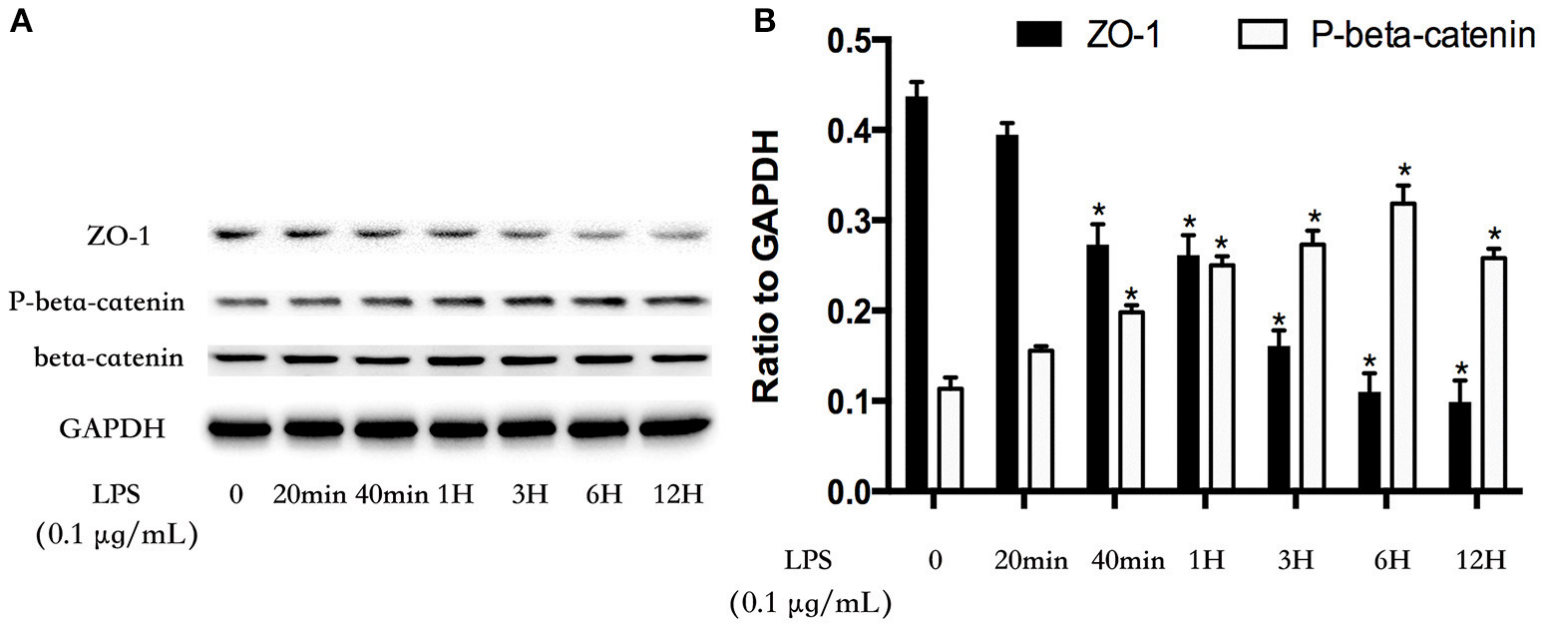
LPS induces degradation of beta-catenin and ZO-1 in HPMECs monolayer. LPS (0.1 μg/ml) induced down-regulation of ZO-1 expression and increase of phosphorylated degradation of beta-catenin in a time-dependent manner **(A)**. The Western blotting results are presented as a histogram showing the band intensity values **(B)**. ^*^P < 0.05 vs. LPS un-treatment group.

**Figure 5:**
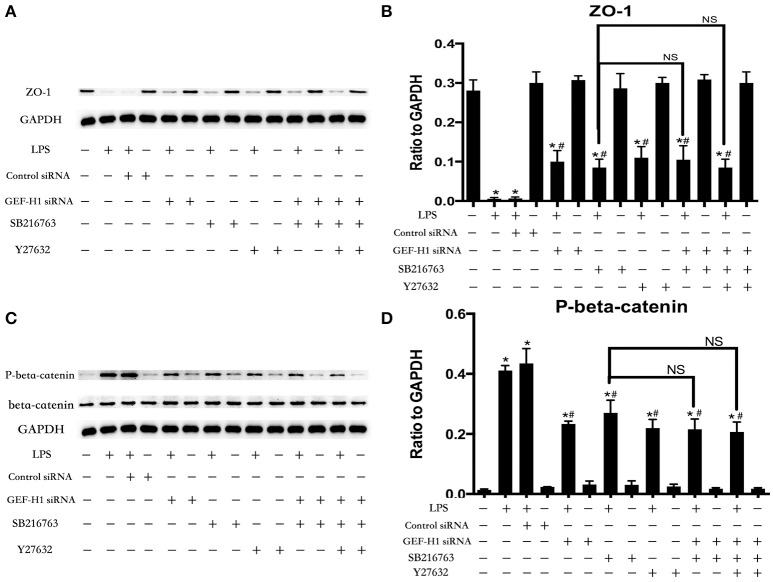
GSK-3beta/GEF-H1/ROCK signaling is required for LPS-induced degradation of beta-catenin and ZO-1. After transfection with GEF-H1 siRNA and Control siRNA for 48 h, HPMECs were treated with SB-216763 (20 μM) and/or Y-27632 (10 μM) for another 1 h prior to LPS stimulation (0.1 μg/ml) for 3 h. The expression of ZO-1 was determined by immunoblotting, and GAPDH protein was used as loading control **(A)**. The Western blotting results are presented as a histogram showing the band intensity values **(B)**. The expression of P-beta-catenin was determined by immunoblotting, and GSK-3beta and GAPDH proteins were used as control **(C)**. The Western blotting results are presented as a histogram showing the band intensity values **(D)**. ^*^P < 0.05 vs. negative control. ^#^*P* < 0.05 vs. corresponding LPS-stimulated group. NS, no significance.

**Figure 6:**
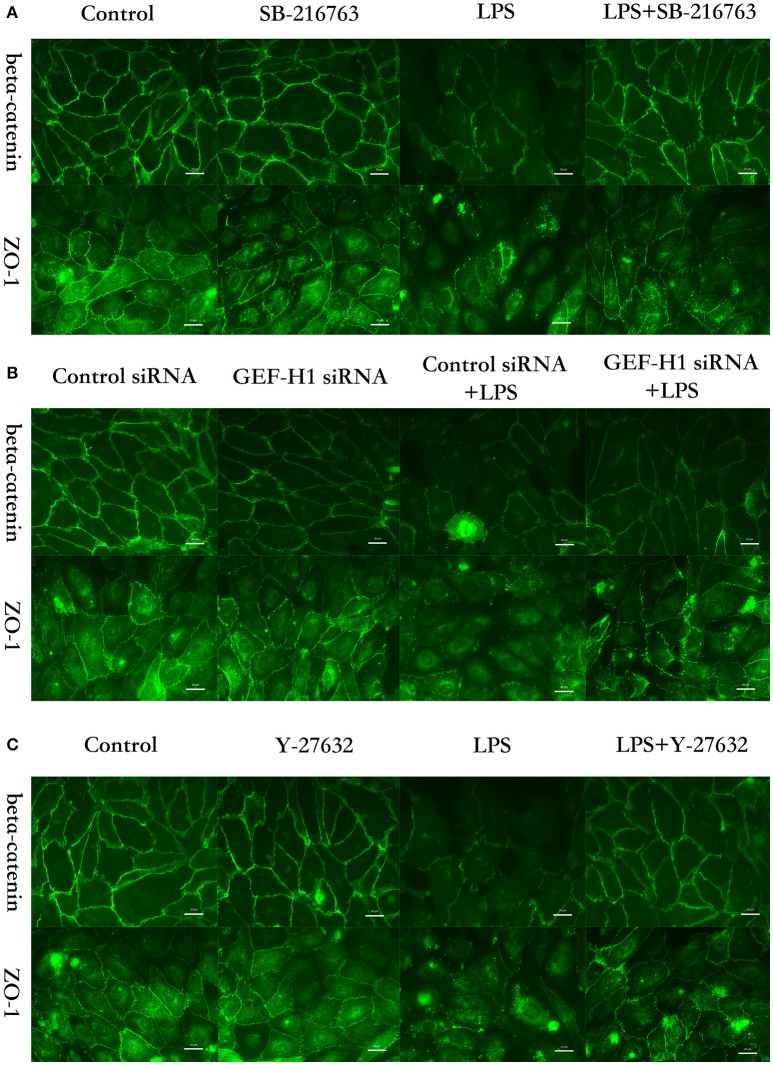
GSK-3beta/GEF-H1/ROCK pathway is involved in LPS-induced HPMECs barrier disruption by beta-catenin and ZO-1. HPMECs monolayer was pretreated with SB-216763 (20 μM) **(A)**, GEF-H1 siRNA **(B)**, or Y-27632 (10 μM) **(C)**, for indicated times and then was exposed to LPS (0.1 μg/ml) for 3 h before fixation and staining with anti-beta-catenin and anti-ZO-1 antibody as described in Materials and Methods. Beta-catenin (green) and ZO-1 (green) were visualized by immunofluorescence microscopy. Red arrows not only represent the expression of beta-catenin and ZO-1 in the membrane of HPMECs but also represent the cell-cell gaps formation in the ECs monolayer.

The cell-cell junctonal protein complexes locate in membrane of contacted vascular ECs (Dejana, 2004). Beta-catenin and ZO-1 are the primary components of AJs and TJs, which play an important role in regulating ECs barrier function. To verify the possibility that LPS-induced GSK-3beta/GEF-H1/ROCK signaling activation exerts its ECs barrier disruptive effect by regulating the expression of beta-catenin and ZO-1. We firstly tested the expression of beta-catenin and ZO-1 after treating with LPS at indicated time points in HPMECs. As a result, LPS rapidly induced the degradation of beta-catenin and ZO-1 (Figures 4A,B). We further pretreated HPMECs with SB-216763, GEF-H1-specific siRNA and Y-27632 (the specific inhibitor of ROCK) before the stimulation of LPS. We found that inhibition of GSK-3beta, GEF-H1 and ROCK significantly reversed LPS-induced degradation of beta-catenin and ZO-1 (Figures 5A–D). Interestingly, we also found that co-inhibition of GSK-3beta and GEF-H1 or GSK-3beta and ROCK produced equivalent inhibitory effects on LPS-induced disruption of beta-catenin and ZO-1. The above data indicates that LPS induced disruption of beta-catenin and ZO-1 via the activation of GSK-3beta/GEF-H1/ROCK signaling pathway. Similarly, the effects of SB-216763, GEF-H1 siRNA and Y-27632 on LPS-induced disruption of beta-catenin and ZO-1 were further confirmed by immunofluorescence staining analysis. Compared with the control group, the LPS group displayed increased cell-cell gaps in ECs monolayer and decreased expression of beta-catenin and ZO-1 in membrane of contacted ECs. However, pretreatment with SB-216763, GEF-H1 siRNA, and Y-216763 all effectively reversed the LPS-induced cell-cell gaps formation and degradation of beta-catenin and ZO-1 (Figures 6A–C).

### GSK-3beta inhibitor attenuates LPS-induced ALI and vascular leak

**Figure 7:**
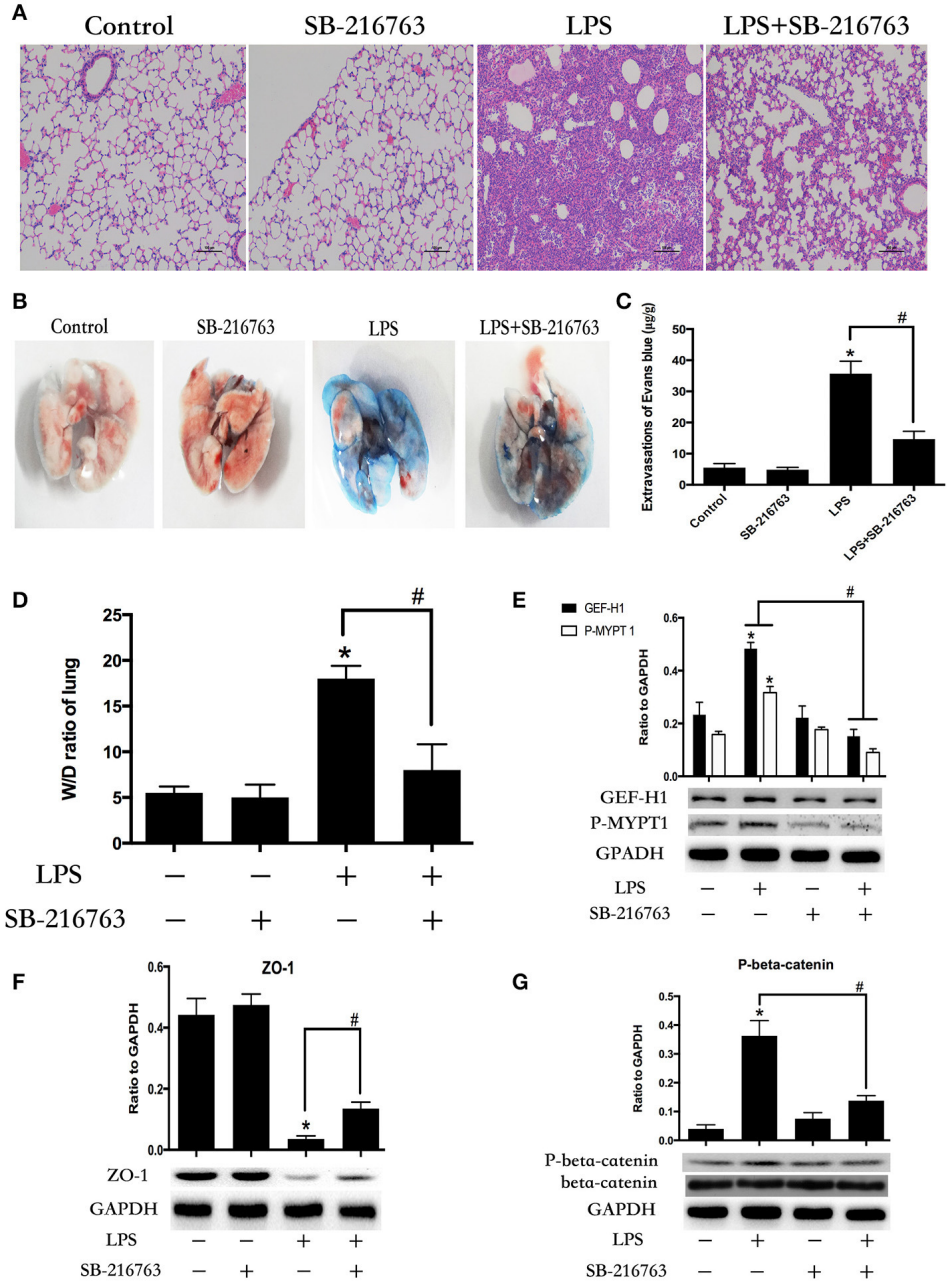
Inhibition of GSK-3beta ameliorates LPS-induced ALI and vascular barrier dysfunction in mice. C57BL/6J mice were challenged with vehicle or LPS (2 mg/kg, i/t) for 24 h with or without SB-216763 pretreatment (20 mg/kg, i/p) (2 h prior to LPS intratracheal instillation) histological analysis of lung tissue by hematoxilin & eosin staining (× 100 magnification) **(A)**. Evans blue dye (30 ml/kg, i/v) was injected 2 h before termination of the experiment **(B)**. The quantitative analysis of Evans blue extravasation was performed by spectrophotometric analysis of Evans blue extracted from the lung tissue samples **(C)**. ^*^P < 0.05 vs. negative control. ^#^*P* < 0.05 vs. corresponding LPS-stimulated group. Wet/dry ratio of lungs from the control, SB-216763 group, LPS group and LPS + SB-216763 group was represented as a histogram according to data **(D)**. Expression of GEF-H1 and P-MYPT 1 in lung tissue samples was evaluated by Western blot **(E)**. Expression of ZO-1 and P-beta-catenin in lung tissue samples was evaluated by Western blot analysis **(F,G)**. ^*^P < 0.05 vs. negative control. ^#^*P* < 0.05 vs. corresponding LPS-stimulated group.

To further determine whether the LPS-induced GSK-3beta activation is required for LPS-induced ALI and vascular leak *in vivo*, the effect of SB-216763 on LPS-induced lung histologic changes and vascular permeability changes were examined in this study. Compared with the control group, intra-tracheal administration of LPS significantly exhibited obvious pathologic changes, including interstitial edema and abundant infiltration of inflammatory cells. However, these changes were ameliorated by pretreatment with SB-216763 (Figure 7A). Effects of GSK-3beta inhibition on LPS-induced lung vascular leak were detected by the Evans blue dye accumulation (Figures 7B,C) and the lung wet-dry weight ratio (Figure 7D) in mice lung. The lung tissues of the mice in the LPS group exhibited obvious Evans blue leak and increase of wet-dry ratio. However, SB-216763 pretreatment obviously inhibited the above changes. SB-216763 treatment also decreased protein levels of beta-catenin, ZO-1, GEF-H1 and ROCK in the lungs from LPS-stimulated mice (Figures 7E–G).

## Discussion

ALI and ARDS are common complications of septic shock in which pulmonary capillary leak is regarded to be the primary initiator (Ware and Matthay, 2000). The integrity of the lung endothelium is an essential requirement for lung function preservation (Barabutis et al., 2016). Several mechanistic studies have revealed that vascular endothelial barrier function is mainly controlled by the stability of intercellular junctions within the EC monolayers (Braga, 2002). However, the underline molecular mechanisms causing the disruption of EC junctional proteins under sepsis are still incompletely understood. GSK-3beta plays a fundamental role in the regulation of inflammation cascade under pathological state (Golpich et al., 2015). However, whether GSK-3beta implicated in the regulation of endothelial barrier function remains poorly defined. In the present study, the results demonstrate that the GSK-3beta/GEF-H1/ROCK signaling pathway plays an important role in LPS-induced HPMECs hyper-permeability via the negative regulation of ZO-1 and beta-catenin expression. In addition, the blockade of GSK-3beta activity effectively ameliorates LPS-induced ALI via inhibition GEF-H1/ROCK activity and disruption of junctional proteins stability in mice lung.

GSK-3beta is implicated in the regulation of several signaling transduction pathways and is a potential therapeutic target for sepsis (Noh et al., 2011). For example, transactivational activity of P65 is decreased by inactivation of GSK-3beta, which inhibits LPS-induced TNF and TF gene expression in monocytes (Guha and Mackman, 2002). P38 is an important determinant for the induction of pro-inflammatory cytokines under sepsis (Chang et al., 2016). LPS-induced activation of P38 was blocked via the knockdown of GSK-3beta (Zhang and Wahl, 2015). Although the inhibitor of GSK-3beta mediated significant protection against endotoxin lethality, the expression and activity of GSK-3beta in LPS-incubated HPMECs is not clear. It is well-known that different phosphorylation sites of GSK3 beta are associated with its activity, and different sites phosphorylation may implicate in different signaling transmission in the same cells after same stimulation. Furthermore, some site phosphorylation may override the other site phosphorylation and play its leading role spatially and temporally. GSK-3beta function is shown to be dependent on tyrosine 216 phosphorylation (Hughes et al., 1993). In primary human brain micro-vascular endothelial cells, de-phosphorylation of GSK-3beta at tyrosine 216 decreased the GSK3-mediated inhibition of beta-catenin (Pinzon-Daza et al., 2014). Some scholars of japan found that the inactivation of GSK-3beta by the phosphorylation of Ser 9 could be reversed by the increased phosphorylation of Tyr 216 on GSK-3beta (Boku et al., 2013). In addition, phosphorylation of GSK-3beta on Tyr216 was increased in cortical neurons associated with ischemia or LPS stimulation (Bhat et al., 2000; Zhao Y. et al., 2014). In the present study, LPS up-regulated the expression of P-GSK-3beta Tyr216 in dose- and time-dependent manners in HPMECs. These data provide evidence that P-GSK-3beta (Tyr216)-mediated activation of GSK-3beta may be implicated in LPS-induced endothelial cell barrier disruption and acute lung injury.

Microtubules (MTs) are major components of the eukaryotic cytoskeleton (Hyman and Karsenti, 1996). MT de-polymerization is closely associated with release and activation of GEF-H1 (Birkenfeld et al., 2008). Our previous studies have verified that LPS rapidly induced MT de-polymerization in human umbilical vein endothelial cells (HUVECs), and when LPS was absent, the MT was under polymerization state (Zhou et al., 2015). The above data suggested that GEF-H1 was inactive before the stimulation of LPS in ECs. Moreover, ROCK is substantially involved in cardio-vascular disorders and leads to tissue factor expression, release of inflammation factors and the breakdown of the endothelial barrier in LPS-stimulated ECs (Noma et al., 2012; Yi et al., 2016). GEF-H1 possessed ROCK-specific enzymatic activity and our previous studies have verified that the GEF-H1/ROCK signaling was abnormal activated and implicated in LPS-induced HUVECs injury (Guo et al., 2012a,b,c; Zhou et al., 2013). Consistent with previous studies, we also observed GEF-H1/ROCK cascade activation in this study, which coincides with the increase in GSK-3beta phosphorylation in LPS-incubated HPMECs. Interestingly, recent studies reported that the MT-associated proteins, key regulators of MT dynamics, were interacted with GSK-3beta and served as its putative substrates (Xu et al., 2015). Activated GSK-3beta leads to a-tubulin deacetylation and MT disassembly (Gassowska et al., 2014). In light of the indirect correlation between GSK-3beta and GEF-H1/ROCK signaling, we further analyzed the role of GSK-3beta on the expression of GEF-H1 and ROCK activity after stimulating with LPS in HPMECs. In the present study, inhibition of GSK-3beta significantly block the LPS-induced up-regulation of GEF-H1 expression and ROCK activation, which indicated that LPS could rapidly activate GSK-3beta/GEF-H1/ROCK signaling pathway in HPMECs. Our previous study has showed that LPS-induced increase of EC monolayers permeability was attenuated by inhibition of GEF-H1/ROCK signaling (Zhou et al., 2013). It remains unknown, however, whether GSK-3beta is involved in LPS-induced ECs hyper-permeability. Inactivation of GSK-3β ameliorated ischemia-induced blood brain barrier (BBB) hyper-permeability (Zhao T. et al., 2014). In this study, LPS-induced decrease of trans-endothelial electrical resistance (TER) was significant reversed by inhibitor of GSK-3beta. Our data suggested that GSK-3beta/GEF-H1/ROCK pathway played an important role in regulation of LPS-induced ECs barrier dysfunction.

In the endothelium, junctional complexes comprise TJs, AJs and gap junctions. The barrier within the vascular membrane is mainly provided by TJs and AJs, among which junctional proteins are composed of multiple trans-membrane proteins and intracellular scaffolding proteins (Bazzoni and Dejana, 2004). Our previous study have showed that GEF-H1/ROCK signaling involved in LPS-induced paracellular hyper-permeability via mediating the expression of TJs and AJs trans-membrane proteins, such as Claudin-1 and VE-cadherin (Zhou et al., 2013). Importantly, the junctional scaffold proteins of TJs and AJs, such as beta-catenin and ZO-1, are essential for barrier formation in human micro-vascular ECs and that it regulates cell-cell tension and cytoskeletal organization (Ben-Ze'ev and Geiger, 1998; Matter and Balda, 2003). However, whether GSK-3beta/GEF-H1/ROCK pathway promoted LPS-induced endothelial hyper-permeability via the regulation of beta-catenin and ZO-1 is not clear.

Phosphorylation of beta-catenin (Ser33/37) targets beta-catenin for ubiquination and degradation by the proteasome (Aberle et al., 1997). Consistent with the previous study (Barton-Pai et al., 2011; Wan et al., 2013), we found that LPS administration evoked increase in the disruption of beta-catenin and ZO-1 in HPMEC monolayers. GSK-3beta has a number of protein substrates. No other substrate has been better characterized to be a primary substrate than beta-catenin (Hsieh et al., 2016). Beta-catenin acts as the substrate of Wnt signaling is degradation by GSK-3beta in resting cells. However, Serving as the main component of adherens junction complex in the endothelial cells, beta-catenin makes its roles more complicated. The endothelial cell adhesion molecule E-cadherin and APC compete for the interaction with beta-catenin (Hulsken et al., 1994). When endothelial cells contacts each other in unstimulated state, beta-catenin will be bound to cadherins and is stabilized and retained at the cell membrane. Mirelle et al have reported that the serine phosphorylation of beta-catenin is required for the strong-to-weak shift of cadherin-based cell adhesion and disruption of cell-cell contacts (Serres et al., 1997). Amy Barton-Pai et al found that Tumor Necrosis Factor-a induces lung vascular hyper-permeability via the activation of GSK-3beta and increased phosphorylation of beta-catenin (Ser33/37) (Barton-Pai et al., 2011). In addition, Ramirez et al. found that GSK-3beta regulated the permeability of BBB under physiologic conditions via ZO-1 (Ramirez et al., 2013). In the present study, suppression of GSK-3beta by specific inhibitor has a direct inhibitory effect on LPS-induced disruption of beta-catenin and ZO-1 in HPMECs. Furthermore, not only co-inhibition of GSK-3beta and GEF-H1 but also co-inhibition of GSK-3beta and ROCK activity did not produce an augmented reverse effect on LPS-induced disruption of beta-catenin and ZO-1 than inactivation of GSK-3beta alone. These data indicated that GSK-3beta as an upstream effector was essential for GEF-H1/ROCK-mediated disruption of ZO-1 and beta-catenin in HPMECs after exposure to LPS.

To gain further insight into the role of the GSK-3beta signaling pathway in lung vascular barrier disruption in LPS-induced ALI, we checked the expression of GEF-H1, beta-catenin, ZO-1 and ROCK activity in the septic murine lung model after inhibition of GSK-3beta. In the present study, GSK-3beta inhibition not only blocked activation of GEF-H1/ROCK pathway but also reversed decrease of beta-catenin and ZO-1 in LPS-stimulated lung tissue. Furthermore, GSK-3beta inactivation protected against LPS-induced lung edema, lung vascular hyper-permeability and ALI. These observations are analogous to previous studies in which authors showed that stiffness-activated GEF-H1/ROCK pathway in lung exacerbates LPS-induced ALI (Mambetsariev et al., 2014). These results indicate that LPS might induce lung vascular endothelial barrier dysfunction-associated ALI through the GSK-3beta/GEF-H1/ROCK signaling pathway *in vivo*.

In summary, our findings in this study point to a specific pathway that GSK-3beta/GEF-H1/ROCK signaling exerts its endothelial barrier disruptive effect by regulating the integrity of beta-catenin and ZO-1 in LPS-induced HPMECs. These data suggest a novel mechanism by which LPS contributes to the initiation of lung micro-vascular disruption and evolution of ALI. In addition, these results identify a critical function for GSK-3beta in modulating micro-vascular barrier disruption-associated ALI *in vivo* in mice and provide a rationale for regulating the lung edema under septic condition. However, even though our study and other studies (Baumgart et al., 2016; Liu et al., 2016) find that there are various conditions in which the possible involvement of GSK-3beta has been implied (pro-inflammation, hyper-coagulation and hyper-permeability), the efficacy of GSK-3beta inhibitor in inhibiting sepsis-associated ALI in humans has not yet been examined successfully, and further investigations are necessary.

## Ethics statement

This study was carried out in accordance with the recommendations of the Declaration of Helsinki. The protocol was approved by the Science and Technology Commission of Shanghai Municipality.

## Author contributions

LY and XH made substantial contributions to analysis and interpretation of data, drafting the article and revising it critically for important intellectual content, and final approval of the version to be published; FG, ZZ, and MC made substantial contributions to conception and design of the work and acquisition of data, editing the manuscript, and final approval of the version to be published; JH made substantial contributions to conception and design of the work and analysis and interpretation of data, drafting and revising the manuscript critically for important intellectual content, and final approval of the version to be published.

### Conflict of interest statement

The authors declare that the research was conducted in the absence of any commercial or financial relationships that could be construed as a potential conflict of interest. The reviewer HW and handling Editor declared their shared affiliation and the handling Editor states that the process nevertheless met the standards of a fair and objective review.
